# Optimization of Railway Mixed Goods Loading Layout considering Stability

**DOI:** 10.1155/2022/1517280

**Published:** 2022-08-21

**Authors:** Juan Wang, Yinghua Yao, Yinggui Zhang, Ximing Wang

**Affiliations:** ^1^School of Logistics and Transportation, Central South University of Forestry and Technology, Changsha 410004, Hunan, China; ^2^School of Traffic and Transportation Engineering, Central South University, Changsha 410075, Hunan, China; ^3^School of Intelligent Equipment Technology, Hunan Vocational College of Science and Technology, Changsha 410004, Hunan, China

## Abstract

It is well known that stability, center-of-gravity balance, and concentrated-weight are key factors of the transportation safety. The reasonable formulation of the loading layout scheme ensures the safety of shipment based on fully utilizing the effective volume and load capacity of freight vehicles. This paper takes the railway mixed goods loading layout as the research object, considering the constraints such as goods loading center-of-gravity balance, the allowable moment of concentrated-weight, supporting and goods placement mode, and taking the maximum comprehensive utilization rates for both effective volume and load capacity of freight vehicle as the optimization objective, an optimization model of railway mixed goods balanced and anticoncentrated-weight loading layout considering stability is built. Additionally, this paper designs mixed goods classified and simple/general goods block composition methods. We improve the representation and selection of layout space, construct goods block selection algorithm based on the greedy d-step lookahead tree search and goods block evaluation function and propose a goods block placement strategy and update rules of layout space after goods block placement. An optimization algorithm of railway mixed goods balanced and anticoncentrated-weight load layout considering stability is designed. The results show that the formulated scheme not only ensures that the goods meet the full support constraints, but also the comprehensive utilization rate of the effective volume and load capacity of the vehicle is not less than 89%, and the probability of meeting the loading center-of-gravity balance and allowable moment of concentrated-weight are as high as 99% and 99.47%, respectively. The proposed method realizes the balanced and anticoncentrated-weight loading of railway mixed goods, ensures the safe, stable, and efficient goods loading, and provides decision support for the safe loading layout of railway goods.

## 1. Introduction

The transportation safety of railway mixed goods of different types, sizes, and weights is closely related to the scientific and reasonable loading layout scheme. How to efficiently prepare the loading layout scheme of railway mixed goods, make full use of the effective volume and load capacity of freight cars, while ensuring the balance of goods loading center of gravity, anticoncentrated-weight, and stability support, has important practical significance for reducing costs and increasing efficiency and safe transportation of railway goods [1].

Many scholars have carried out some research related to goods loading layout problems, mainly focusing on optimization objectives, constraints, goods combination methods, spatial representation, loading layout algorithm design, etc. For optimization objectives and constraints, Huang et al. for the single container loading problem, with the objective of minimizing the length of occupied space in a container, used a 0–1 mixed integer linear programming model to describe the problem and proposed a load distribution heuristic algorithm [2]. Costa et al. divided the center-of-gravity balance constraint into three subconstraints: longitudinal, transverse, and vertical [3]. Ramos et al. proposed a variety of group biased random key genetic algorithms based on static mechanical equilibrium conditions [4], and considering the limitation of loading efficiency, a physical loading order algorithm is proposed [5]. In terms of the way the goods are combined, Bortfeldt et al. combined goods into vertical layers and proposed a hybrid genetic algorithm for strongly heterogeneous goods [6]. Zhu Xiang et al. constructed goods as towers and proposed an optimization method for loading multiple pieces of concentrated-weight goods in one vehicle [7]; however, for high-density nonconcentrated-weight goods, when the distribution of goods is unreasonable, it will also produce concentrated-weight. In addition, aiming at the problem of balanced loading of multiple vehicles and multiple pieces of goods, they put forward the idea of symmetrical loading of multiple vehicles and algorithm optimization strategy [8]. Fanslau et al. extended the way of goods combination and proposed a general block generation method [9]. In terms of spatial representation, Jiang Yidong et al. proposed the method of using a trinomial tree data structure to represent the partitioning of rectangular goods layout space by putting a suitable goods block in the lower left corner of each small space of the partition [10]. Moura et al. proved that using the maximum coverage method to represent the layout space can obtain better solutions than the partitioning method [11]. In addition, there are many studies on load layout algorithms and strategies. Araya et al. determined the parameters of goods evaluation function by the control variable method to improve the efficiency of goods unit search [12]. Wang Zhe et al. constructed and designed an optimization model and algorithm for the similar goods [13]. Liu Xiaoqun et al. constructed and designed an optimization model and algorithm based on different benchmarks for multivariety goods [14]. Bischoff proposed a constructive heuristic embedding search algorithm and placement rules for the packing problem of goods with different bearing strength [15]. Lei Dingyou et al. proposed a mixed and balanced loading method of railway containers for light and heavy goods based on the central skeleton idea, but they did not include the load capacity utilization rate into the optimization objective [16]. Huang et al. proposed a novel technique that combines a differential evolution algorithm with a ternary search tree model to solve the three-dimensional container loading problem [17]. Zhang et al. proposed an optimization method for balanced loading layout of railway container mixed goods, but stability constraints such as full support are not involved [18].

Existing studies have provided strong support for the loading layout of railway mixed goods. However, most of them only take the volume of the loading space as the optimization objective, ignoring the optimization of the load capacity utilization. In addition, the constraints related to concentrated-weight and stability are less considered. Motivated by the above considerations, to reflect the utilization rate of freight vehicles more objectively and comprehensively, and ensure the loading efficiency and safety, this paper studies the balanced and anticoncentrated-weight loading layout of railway mixed goods considering stability. Specifically, this problem uses railway freight vehicles (such as gondola cars and boxcars) to load mixed goods of different types, sizes, and weights, with the optimization goal of maximizing the comprehensive utilization of effective volume and load capacity of freight vehicles, and considering the practical constraints such as loading center-of-gravity balance, the allowable moment of concentrated-weight and full support. For this problem, based on the process of goods loading layout, we designed algorithms and rules such as mixed cargo classification, fully supported goods block unit generation, goods block unit selection and placement, and update of remaining available layout space. Furthermore, an optimization method of railway mixed goods balanced and anticoncentrated-weight load layout considering stability is proposed. Finally, the feasibility and effectiveness of the method are verified by combining the international standard example and the mixed goods example generated based on the standard example.

The rest of the paper is organized as follows. In Section 2, the problem is defined, the practical constraints involved in loading are analysed, and the mathematical model of the problem is established.  Section 3 designs a heuristic algorithm for railway mixed goods balanced and anticoncentrated-weight loading layout optimization considering stability based on the six elements of goods loading layout. In Section 4, the test and comparative analysis of the international standard example and the improved mixed goods examples are carried out. Finally, conclusions are drawn in Section 5.

## 2. Problem Description and Mathematical Model

### 2.1. Problem Description

**Figure 1:**
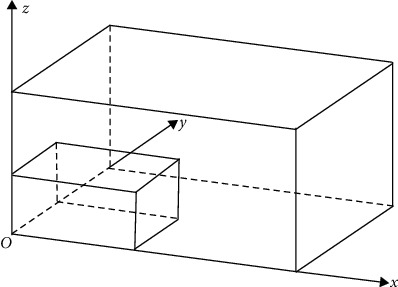
Space rectangular coordinate system inside the freight vehicle.

Given a set of rigid cuboid goods set **C**={**C**_1_,…, **C**_**i**_,…, **C**_**n**_} with uniform density of class *n*, whose total volume and total weight are *V*_*n*_ and *Q*_*n*_, respectively; denote by **C**_**i**_={*c*_*i*1_,…, *c*_*ij*_,…, *c*_*iu*_} the goods of class *i* containing *u* goods, the volume of class *i* goods is *V*_*i*_, and the weight of class *i* goods is *Q*_*i*_; *l*_*ij*_, *w*_*ij*_, *h*_*ij*_, *v*_*ij*_, *q*_*ij*_ indicate the length, width, height, volume, and weight of individual goods, respectively. Assuming that the effective space inside the vehicle is rectangular, and the goods can be loaded and stacked, not special cargo such as dangerous goods, allowed to have surplus to be left for the next assembly. There is no loading and unloading operation on the way, and the goods all arrive at the station together [19]. Take the left front inner lower corner of the vehicle as the coordinate origin *O*, take the floor of the vehicle as the X-Y plane, and establish the space rectangular coordinate system in Figure 1.

### 2.2. Constraint Analysis

Set *L*, *W*, *H*, *V* for the internal length, width, height, and effective volume of the railway vehicle, respectively, *Q*, *Q*_v_ for the maximum allowable weight and self-weight of the vehicle, respectively, *D* for the center distance of bogie, *H*_v_ for the vehicle floor to rail height, and *H*_o_ for the empty vehicle center of gravity high. In order to ensure transport safety, in accordance with the current “Rules for railway cargo loading and reinforcement” [20], the longitudinal deviation of the combined center of gravity after loading relative to the intersection of the vertical and horizontal center line of the vehicle floor shall be calculated according to Δ_1_=min{(*Q* − *Q*_*C*_) × *D*/2*Q*_*C*_, 5  *D*/*Q*_*C*_}, where *Q*_*C*_=∑_*i*=1_^*n*^∑_*j*=1_^*u*^*μ*_*ij*_*q*_*ij*_ for the total weight of the loaded goods, *μ*_*ij*_ for 0 or 1 that the goods *c*_*ij*_ is not loaded or has been loaded; the lateral deviation Δ_2_ ≤ 100*mm*; the height of the center of gravity of the vehicle from the rail surface is Δ_3_ ≤ 2000*mm*, more than that should be speed limit [20].

Using the X-Y plane rectangular coordinate system of Figure 1, setting (*G*_1_, *G*_2_, 0) for the coordinates of the intersection *G* of the vehicle floor longitudinal and transverse center line and (*c*_*ij*_^*x*^, *c*_*ij*_^*y*^, *c*_*ij*_^*z*^) for the coordinates of the center of gravity of the goods *c*_*ij*_ after it is put into the vehicle. Then, the coordinates (*G*_*x*_,  *G*_*y*_,  *G*_*z*_) of the goods combined center of gravity in the vehicle are (∑_*i*=1_^*n*^∑_*j*=1_^*u*^*μ*_*ij*_*c*_*ij*_^*x*^*q*_*ij*_/*Q*_*C*_, ∑_*i*=1_^*n*^∑_*j*=1_^*u*^*μ*_*ij*_*c*_*ij*_^*y*^*q*_*ij*_/*Q*_*C*_, ∑_*i*=1_^*n*^∑_*j*=1_^*u*^*μ*_*ij*_*c*_*ij*_^*z*^*q*_*ij*_/*Q*_*C*_). The actual longitudinal deviation of the combined center of gravity after loading is *S*_1_=|*G*_*x*_ − *G*_1_|, the transverse deviation is *S*_2_=|*G*_*y*_ − *G*_2_|, and the height of the center of gravity of the vehicle is *S*_3_= *G*_*z*_+*H*_v_. When *S*_1_ ≤ Δ_1_, *S*_2_ ≤ Δ_2_, *S*_3_ ≤ Δ_3_, the goods loading center-of-gravity balance constraint is satisfied.

**Figure 2:**
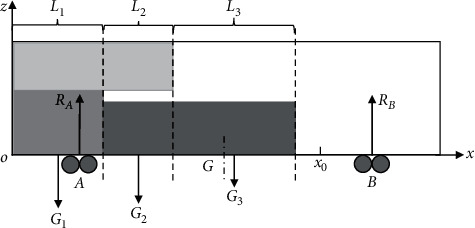
Diagram of longitudinal stress on vehicle floor.

Using the X-Z plane rectangular coordinate system of Figure 1, the balance or not of the forces in the length direction of the vehicle is the main reason whether the concentrated-weight is generated or not [7]. Here, only the force in the length direction of the vehicle is analysed, as shown in Figure 2, the front and rear bogies are supported by *R*_*A*_ and *R*_*B*_ and the coordinate positions are *X*_*A*_ and *X*_*B*_, respectively. According to the cargo boundary, the vehicle floor is divided into *N* layers *L*_1_, *L*_2_, *L*_3_… *L*_*N*_ that is perpendicular to the longitudinal center line, *G*_1_, *G*_2_, *G*_3_… *G*_*N*_ are the corresponding goods combined gravity of each layer, respectively, and *X*_1_, *X*_2_, *X*_3_… *X*_*N*_ are the distances from each combined gravity force to any position *x*_0_ on the floor length. According to the moment balance equation, the point *B* as a balance point to find out the vehicle floor in *x*_0_ position bending moment *M*_0_ is *R*_*A*_ · *X*_*A*_ − ∑_*l*=1_^*N*^*G*_*l*_ · *X*_*l*_. For different loading schemes, the maximum working bending moment *M*_max_ of the vehicle floor and its coordinate position can be determined according to the above method. When not more than the allowable bending moment [*M*], that is, *M*_max_ ≤ [*M*] is to meet the concentrated-weight allowable bending moment constraint.

Loading stability generally requires that the goods in the space can be placed stably without tilting and collapsing, mostly by restricting the supported area of the bottom surface of the goods in the vertical direction, and limiting the supported area of the bottom surface of the goods not less than the specified proportion of its own bottom surface areas, such as 55%, 75%, or 100%, that is, partial support constraint or complete support constraint. In this paper, the full support constraint is adopted to ensure that the bottom surface of the goods is fully supported by the vehicle floor or other loaded goods.

### 2.3. Model Construction

In summary, this paper considers the goods loading center-of-gravity balance, the allowable moment of concentrated-weight, full support, and other constraints, with the maximum comprehensive utilization rates for both effective volume and load capacity of freight vehicle as the optimization objectives. Finally, the optimization model of railway mixed goods balanced and anticoncentrated-weight loading layout considering stability are built.

(1) $$\begin{array}{c} \max Z=\frac{\alpha \sum_{i=1}^{n}\sum_{j=1}^{u}\mu_{ij}l_{ij}w_{ij}h_{ij}}{V}+\frac{\beta \sum_{i=1}^{n}\sum_{j=1}^{u}\mu_{ij}q_{ij}}{Q}, \end{array}$$

s.t.

(2) $$\begin{array}{c} S_{1}\leq \Delta_{1}, \end{array}$$

(3) $$\begin{array}{c} S_{2}\leq \Delta_{2}, \end{array}$$

(4) $$\begin{array}{c} S_{3}\leq \Delta_{3}, \end{array}$$

(5) $$\begin{array}{c} M_{\max}\leq \left[M\right], \end{array}$$

(6) $$\begin{array}{c} l_{ij}^{*}\leq c_{ij}^{x}\leq L-\left(l_{ij}-l_{ij}^{*}\right), \end{array}$$

(7) $$\begin{array}{c} w_{ij}^{*}\leq c_{ij}^{y}\leq W-\left(w_{ij}-w_{ij}^{*}\right), \end{array}$$

(8) $$\begin{array}{c} h_{ij}^{*}\leq c_{ij}^{z}\leq H-\left(h_{ij}-h_{ij}^{*}\right), \end{array}$$

(9) $$\begin{array}{c} \left|c_{ij}^{x}-c_{st}^{x}\right|\geq \frac{\left(l_{ij}+l_{st}\right)}{2}, \end{array}$$

(10) $$\begin{array}{c} \left|c_{ij}^{y}-c_{st}^{y}\right|\geq \frac{\left(w_{ij}+w_{st}\right)}{2}, \end{array}$$

(11) $$\begin{array}{c} \left|c_{ij}^{z}-c_{st}^{z}\right|\geq \frac{\left(h_{ij}+h_{st}\right)}{2}, \end{array}$$

(12) $$\begin{array}{c} \left(\left(\left(l_{ij}^{x},w_{ij}^{y},h_{ij}^{z}\right)=\left(l_{ij},w_{ij},h_{ij}\right)\right)\right.\vee , \end{array}$$

(13) $$\begin{array}{c} S_{ij}=l_{ij}^{x}w_{ij}^{y}\cdot 100\%, \end{array}$$

(14) $$\begin{array}{c} \sum_{i=1}^{n}\sum_{j=1}^{u}\mu_{ij}l_{ij}w_{ij}h_{ij}\leq V, \end{array}$$

(15) $$\begin{array}{c} \sum_{i=1}^{n}\sum_{j=1}^{u}\mu_{ij}q_{ij}\leq Q,i,s\in \left\{1,2,3,\ldots ,n\right\};j,t\in \left\{1,2,3,\ldots ,u\right\}, \end{array}$$

where (*l*_*ij*_^*∗*^, *w*_*ij*_^*∗*^, *h*_*ij*_^*∗*^) is the position of the center-of-gravity of goods *c*_*ij*_, that is, its geometric center, (*x*_*ij*_, *y*_*ij*_, *z*_*ij*_) is the coordinates of the vertex of the left front lower corner of *c*_*ij*_ after it is put into the vehicle, and *l*_*ij*_^*x*^, *w*_*ij*_^*y*^, *h*_*ij*_^*z*^ is the dimensional length of the projection in the direction of *x*, *y*, *z* axis. Formula (1) is the objective function, which indicates maximizing the comprehensive utilization rates for both effective volume and load capacity of freight vehicle, such that *α* + *β* = 1, 0 ≤ *α* ≤ 1,0 ≤ *β* ≤ 1, *α* = *Q*_*vw*_′/2*Q*_*vw*_, where the volume-weight of the vehicle is *Q*_*vw*_ = *Q*/*V*, the volume-weight of the loaded goods is *Q*_*vw*_′ = ∑*q*_*ij*_/∑*μ*_*ij*_*l*_*ij*_*w*_*ij*_*h*_*ij*_. Formulas (2)–(4) are the goods loading center-of-gravity balance constraints; formula (5) is the allowable moment of concentrated-weight constraint; formulas (6)–(8) indicate that the loaded goods do not exceed the vehicle boundary constraint; formulas (9)–(11) indicate that any two goods *c*_*ij*_, *c*_*st*_ cannot overlap each other constraints; formula (12) for the placement of goods constraints; formula (13) for the full support constraints; formula (14) for the vehicle effective volume constraints; and formula (15) for the vehicle maximum allowable load constraints.

## 3. Algorithm Design

The studied problem is an NP-hard packing problem. Based on the six elements of goods loading layout [21], focusing on the internal space and goods of freight vehicle, the classification of mixed goods and the construction method of goods blocks, the selection and placement strategy of goods blocks are designed, the representation, selection and update rules of layout space are given, and a heuristic algorithm for railway mixed goods balanced and anticoncentrated-weight loading layout optimization considering stability is formed.

### 3.1. Layout Space Representation

**Figure 3:**
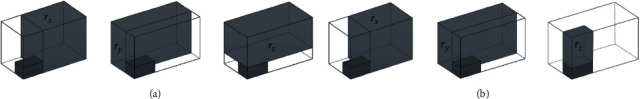
Layout space represented by maximum coverage method.

Using the maximum coverage method [11]to represent the layout space, as shown in Figure 3(a), after the goods are put into the space, three great rectangular layout spaces *r*_*x*_, *r*_*y*_, *r*_*z*_ are generated in the three directions of *x*, *y*, *z*, respectively. Considering the stability constraint, the bottom of all the generated layout spaces should be fully supported by the top surface of the placed goods or the floor of the vehicle. Therefore, it is kept unchanged in the *x* and *y* directions, while a rectangular space determined by the support area of the top of the loaded goods is generated in the *z* direction, as shown in Figure 3(b).

### 3.2. Goods Classification and Goods Blocks Construction

Constructing goods block is currently the most effective way to solve the packing problem [22]. The properties of mixed goods are complex. Taking the bubble weight ratio of goods 1: *g*_1_ and 1: *g*_2_ as the boundary, combined with the weight, volume, and density of goods, the mixed goods are divided into heavy goods, middle goods, and light goods. On this basis, different types of goods blocks are generated. Specific details are described in Algorithm 1.

Blocks can be divided into simple blocks and general blocks. Simple block is a goods block composed of identical goods. The details are as shown in Algorithm 2.

The types and placement methods of goods constituting the general block are not limited, and gaps are allowed in the block, but the ratio of the total volume of goods contained in the block to the minimum external cuboid volume of the block shall meet certain requirements, and the bottom of all goods in the block shall be fully supported by the top surface of goods or the bottom of the block to meet the stability constraints. The specific process is shown in Algorithm 3.

**Figure 4:**
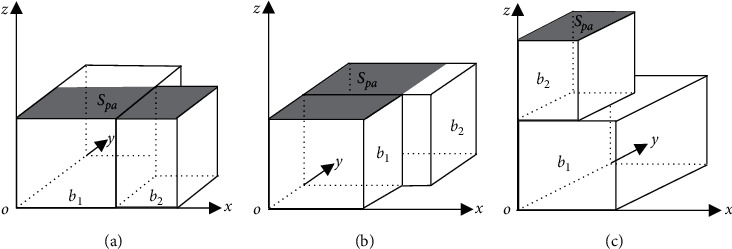
Diagram of general block construction conditions.

Where block *b* is constructed under the condition that block *b*_2_ and block *b*_1_ should be of equal height when combined along the *x*-axis and *y*-axis directions of block *b*_1_, respectively, as shown in Figure 4(a), and that the length of the rectangular area region is *S*_*pa*_ where the upper surface of the block that can provide effective support is *S*_*l*_=*l*_*b*_1__+*l*_*b*_2__, and the width is *S*_*w*_=min(*w*_*b*_1__, *w*_*b*_2__) when along the *x*-axis. As shown in Figure 4(b), the length of *S*_*pa*_ is *S*_*l*_=min(*l*_*b*_1__, *l*_*b*_2__) and the width is *S*_*w*_=*w*_*b*_1__+*w*_*b*_2__ when along the *y*-axis; when combined along the *z*-axis direction of block *b*_1_, as shown in Figure 4(c), the conditions that should be satisfied are *l*_*b*_1__ ≥ *l*_*b*_2__, *w*_*b*_1__ ≥ *w*_*b*_2__, the length of *S*_*pa*_ is *S*_*l*_=*l*_*b*_2__ and the width is *S*_*w*_=*w*_*b*_2__.

### 3.3. Selection of Layout Space

**Figure 5:**
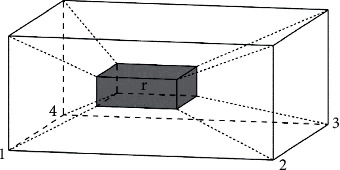
The Manhattan distance between the layout space and each vertex of the freight vehicle.

As shown in Figure 5, selecting the layout space by Manhattan distance [21]. Then, we set the coordinates of the vertex of a layout space *r* in the vehicle as(*r*_*x*_,  *r*_*y*_,  *r*_*z*_), and the coordinates of the corresponding vehicle vertex as (*R*_*x*_,  *R*_*y*_,  *R*_*z*_), then the Manhattan distance between the two points is |*R*_*x*_ − *r*_*x*_|+|*R*_*y*_ − *r*_*y*_|+|*R*_*z*_ − *r*_*z*_|. Considering the stability constraints, it should be ensured that the goods block is placed on the bottom of the fully supported layout space. Therefore, only the Manhattan distance between the four corners of the bottom of the layout space and the four corners of the vehicle bottom should be considered, and the goods block should be placed at the bottom corner of the space corresponding to the shortest Manhattan distance.

### 3.4. Selection of Goods Blocks

Using *V*(*b*) as the volume of goods block *b*, *C*(*b*, *p*) as the direct contact or indirect projection coverage, *L*(*b*, *r*) as the spatial loss rate [16], *N*(*b*) as the number of goods contained in the goods block, and *W*(*b*) as the weight of the goods block, then the goods block selection evaluation function of formula (17) is constructed. When the parameter *λ*`*p*`*φ*`*γ*`*δ*`*ε* takes a suitable value, the higher the value of the evaluation function, the more likely the goods block is selected in preference.

(17) $$\begin{array}{c} f\left(b,r\right)=V{\left(b\right)}^{\lambda }\cdot C{\left(b,p\right)}^{\varphi }\cdot {\left(1-L\left(b,r\right)\right)}^{\gamma }\cdot N{\left(b\right)}^{-\delta }\cdot W{\left(b\right)}^{\varepsilon }. \end{array}$$

**Figure 6:**
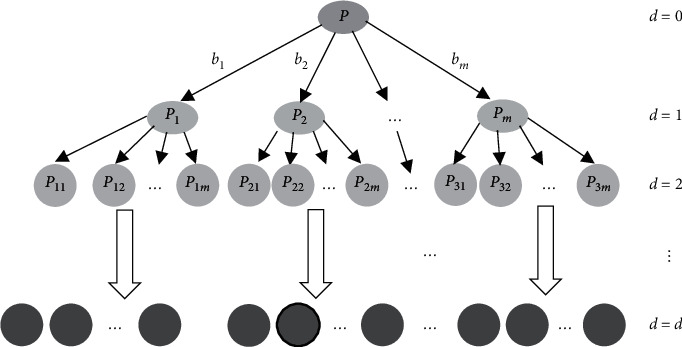
Diagram of cargo block selection based on greedy d-step lookahead tree search.

In order to load the local optimal goods block as much as possible, based on the greedy d-step lookahead tree search, the goods block is selected with formula (17) as the evaluation function, and the goods block selection process is constructed as Algorithm 4. In Figure 6, if the bold path is the optimal layout scheme, the local optimal goods block that should be put into the current *r* is *b*_2_.

### 3.5. Placement of Cargo Blocks and Updating of Layout Space

Firstly, place the heavy goods that have a great impact on the position of the total center of gravity, place the heavy goods close to the corner of the vehicle, and then place the middle goods and light goods. Load the goods in the order from the corner of the vehicle to the center, so that the final remaining debris space is concentrated in the center of the vehicle, and the goods with large weight or density are distributed near both sides of the vehicle bogie, so as to reduce space waste, ensure the balance of center of gravity, and avoid concentrated-weight. When the goods are placed, there is no available space or the total weight of goods exceeds the vehicle maximum allowable load constraints, so the layout shall be ended.

When a goods block is placed into a space, a new layout space is created, and the space needs to be updated to delete the original space, the duplicate space, the contained small space, and the part where the goods block crosses and overlap with the existing space, and keep or create the remaining available and fully supported space. Since the existing spaces all satisfy the stability constraint, there are 50 possible cases of cross overlap between goods blocks and spaces: 4, 8, 5, and 1 cases of 1, 2, 4, and 8 vertices of goods blocks in spaces; 8, 5, and 1 cases of 2, 4, and 8 vertices of spaces in goods blocks; 12, 3 cases of goods blocks running along the boundary of spaces along the direction of coordinate axes and from the middle of spaces, respectively; 3 cases where the goods block divides the space into two parts.

### 3.6. Overall Algorithm for Loading Layout

The above process is generally adjusted and optimized by introducing an indicator *t*, *t*=∑_*n*_*u*/*n* to distinguish the goods structure. When *t* > 6, it is a weakly heterogeneous problem, and a simple block is constructed. When *t* ≤ 6, it is a strongly heterogeneous problem, then a general block is constructed. When the computation time is left, a doubled greedy d-step lookahead tree search is performed. To sum up, the overall flow of railway mixed goods balanced and anticoncentrated-weight loading layout optimization considering stability is shown in Algorithm 5.

## 4. Example Analysis

To verify the validity and feasibility of the proposed method, 1600 international standard arithmetic cases used to evaluate the loading layout problem are adopted [23], on which the goods weight is considered, and the range of goods density is set as (*Q*_*T*_/2*V*_*T*_, *Q*_*T*_(1/*E*[*x*]+1)/2*V*_*T*_), *Q*_*T*_/*V*_*T*_ is the average density of the goods, *E*[*x*] is the beta distribution expectation, and the goods density of the arithmetic cases is obtained according to *f*(*x*, 2,5)[24]. The arithmetic cases are numbered from BR0-BR15 with 16 groups, each group has 100 arithmetic cases, and the goods types are increased from 1 class of BR0 to 100 classes of BR15, where BR0 is a homogeneous class problem, BR1-7 is a weakly heterogeneous class problem, and BR8-BR15 is a strongly heterogeneous class problem. *P*_60_ vehicle is selected for shipment, and its relevant attribute parameters are as follows: *D*  = 11500 mm, *H*_v_  = 1144 mm, *H*_o_  = 1315 mm, *Q*  = 60 t, *Q*_v_  = 22.2 t, *L*  = 15470 mm， *W*  = 2830 mm, *H*  = 2750 mm, and *V*  = 120 m^3^. Since there is no unified standard for the soak weight ratio of land goods, referred to the standard of soak weight ratio of other transportation modes and combined with the actual railway transportation, 300 *kg*/*m*^3^ and 167 *kg*/*m*^3^ are adopted to *g*_1_ and *g*_2_ in the Algorithm 1, respectively. The values of parameters *λ* and *ε* in the selection evaluation function for the goods block are determined to be 0.4 and 0.2, respectively, using the control variable method; and the other parameter values are referred to the work studied by Araya et al. [8], which are *φ*=4,  *γ*=1,  *δ*=0.2,  *p*=0.04. Moreover, the search tree depth factor of d=2 is used to provide a better trade-off between search accuracy and running time. The average running time of 1600 cases is approximately 100 s (±10*s*), the test computer processor is Intel(R) Core(TM) i7-8565U CPU @1.80 GHz, the running memory is 8 GB, the operation system is Windows 10 (64-bit). And then the algorithm is implemented by Java language (JDK version 1.8, 64-bit), which is compiled by Eclipse software (Oxygen version).

### 4.1. Comparative Analysis of International Standard Examples

Based on the above, the scenarios of simple and general blocks are constructed by the test standard cases, and the results of that are shown in Table 1. The results are the average results of the same group including 100 cases, “Vol” is the effective volume utilization rate, “*Wt*” is the load capacity utilization rate, “Sum” is the comprehensive utilization rate, “*AV*” is the average of the results of certain groups of cases, “*Unb*” is the number of cases that dissatisfy the constraint of loading center-of-gravity balance, and “*WtC*” is the number of cases that dissatisfy the allowable moment of concentrated-weight constraint.

By comparing and analyzing the results of columns *SB* and *GB*, it can be seen that the overall results in constructing general blocks are better than those of simple blocks, and the average volume utilization rate, load capacity utilization rate, and comprehensive utilization rate of the proposed method can reach more than 92%, 87%, and 89%, respectively. From “*Unb*” and “*WtC*”, it can be inferred that the satisfaction rate of the loading center-of-gravity balance constraint can meet 99.8%, and the satisfaction rate of the concentrated-weight allowable bending moment constraint can reach to 99.87%, so the loading effect is beneficial enough to meet the actual loading. In addition, for BR0-BR7, the results are better than general blocks when constructing simple blocks, which is the opposite for BR8-BR15. Thus, judging the goods structure type by indicator *t* to construct the corresponding goods blocks can combine advantages to make better loading results.

### 4.2. Comparative Analysis of Mixed Goods Examples Generated Based on Standard Examples

To further verify the effectiveness of the proposed method for mixed goods with greater differences in density, volume, and weight, we increase the goods size, weight interval, and beta distribution on the basis of international standard cases, and generate mixed goods examples. Simple blocks are constructed for BR0-BR7 and composite blocks are constructed for BR8-BR15. The test is carried out with and without consideration of stability.

From the results of Table 1, it is obvious that for the mixed goods examples considering stability, the average volume utilization rate, load utilization rate, and comprehensive utilization rate of the proposed method are lower than the standard examples, while they can reach more than 91%, 86%, and 89% respectively, and the satisfaction rate of the loading center-of-gravity balance constraint and the concentrated-weight allowable bending moment constraint can meet 99% and 99.47%, respectively. The average volume utilization rate, load capacity utilization rate, and comprehensive utilization rate are slightly higher for the mixed goods examples without considering the stability, but the satisfaction rate of the load center-of-gravity balance constraint and the allowable bending moment constraint of the concentrated-weight is much worse, so the comparison shows that the proposed method can achieve favorable loading effects on basis of ensuring the safety and stability of mixed goods transportation.

## 5. Conclusion

1. In this paper, several factors are considered, such as the balance of the combined center of gravity after goods loading, concentrated-weight and full support, and the effective volume and load capacity of the freight vehicle are included in the optimization objective. Then the problem optimization model is constructed. Next, we designed algorithms and rules such as mixed cargo classification, fully supported goods block unit generation, goods block unit selection and placement, and update of remaining available layout space. Furthermore, an optimization method of railway mixed goods balanced and anticoncentrated-weight load layout considering stability is proposed.
2. The proposed method not only meet the goods loading stability but also ensure the comprehensive utilization rate of effective volume and load capacity of the vehicle which is not less than 89%, loading center-of-gravity balance, concentrated-weight allowable bending moment constraints to meet the probability of up to 99% and 99.47%. The method effectively realizes the railway mixed goods balanced and anticoncentrated-weight efficient loading, ensures the safety of railway goods shipment, and provides decision-making reference for railway goods loading layout.
3. The problem of railway goods loading layout is complex and diverse. For simplicity, the interior of the freight vehicle is assumed to be a cuboid space, and the loading layout is carried out from the angle of the loading space. In the future, the problem algorithm should be further improved by considering the specific loading door position and shape attributes of the vehicle.

## Figures and Tables

**Algorithm 1:**
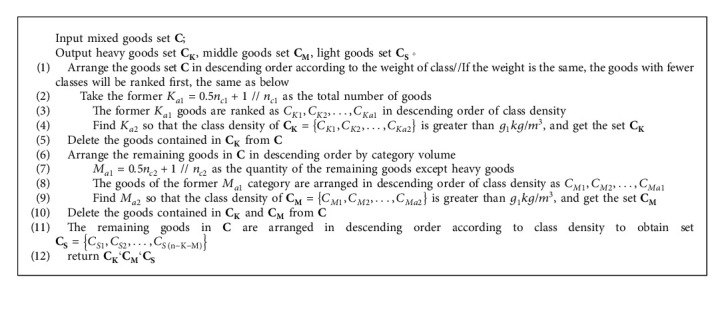
Mixed goods classification algorithm.

**Algorithm 2:**
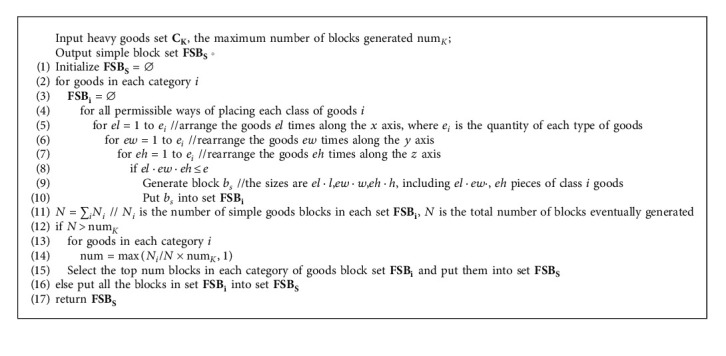
Simple block construction algorithm (Taking heavy goods set as an example).

**Algorithm 3:**
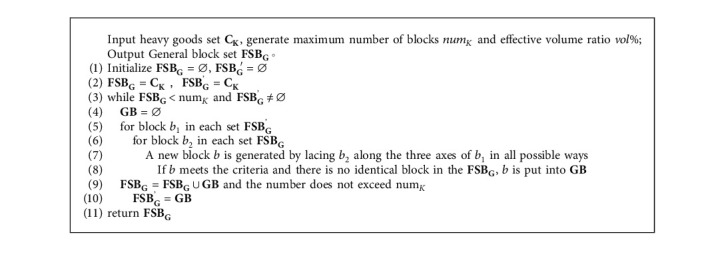
General block construction algorithm (Taking heavy goods set as an example).

**Algorithm 4:**
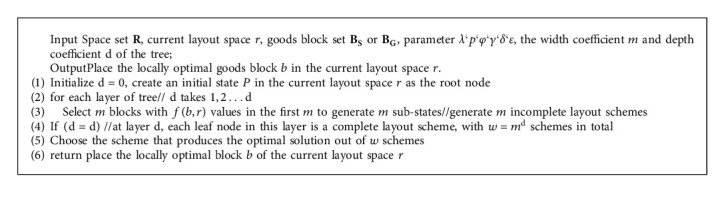
Goods block selection algorithm.

**Algorithm 5:**
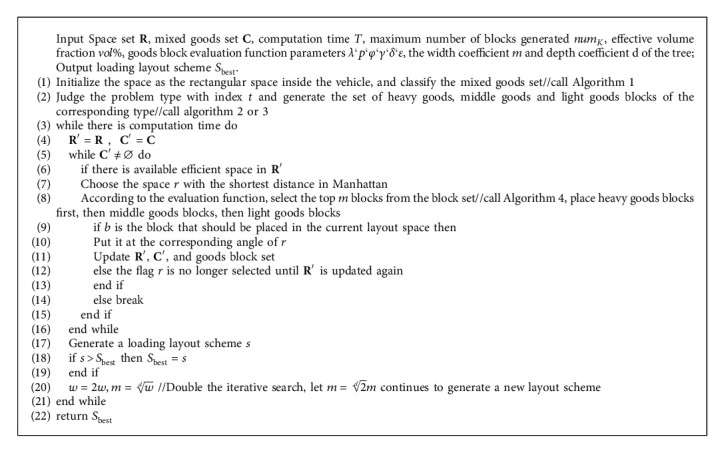
Loading layout optimization overall algorithm.

**Table 1:** Test results under different examples and conditions.

Examples		International standard goods calculation examples										Mixed goods calculation examples (improved on the basis of international standard calculation example)									
		*SB* (Simple block, consider stability)					*GB* (General block, consider stability)					Construct *SB* or *GB*from *t* (consider stability)					Construct *SB* or *GB* from *t* (regardless of stability)				
		Vol. (%)	*Wt*. (%)	*Sum*. (%)	*Unb*. (/)	*WtC*. (/)	Vol. (%)	*Wt*. (%)	*Sum*. (%)	*Unb*. (/)	*WtC*. (/)	Vol. (%)	*Wt*. (%)	*Sum*. (%)	*Unb*. (/)	*WtC*. (/)	Vol. (%)	*Wt*. (%)	*Sum*. (%)	*Unb*. (/)	*WtC*. (/)
Homogeneous	BR0	89.67	82.35	85.86	1	1	89.90	82.17	85.86	2	2	88.82	81.96	85.25	1	1	89.59	82.34	85.81	5	5
Weak heterogeneous	BR1	91.52	82.80	86.94	0	0	91.22	82.62	86.71	1	1	90.06	82.19	85.95	3	2	91.33	82.89	86.90	4	3
	BR2	91.74	83.75	87.56	0	0	91.33	83.57	87.28	0	0	90.92	83.34	86.97	2	2	91.89	83.76	87.64	9	4
	BR3	92.59	84.80	88.52	0	0	92.09	84.82	88.31	0	0	92.05	84.30	88.00	3	1	92.12	84.43	88.11	7	5
	BR4	92.96	84.74	88.66	1	1	92.52	84.60	88.38	2	1	92.29	85.13	88.57	1	0	92.86	85.69	89.13	3	0
	BR5	93.14	85.92	89.38	2	1	92.85	85.73	89.15	0	0	92.43	85.36	88.75	0	0	93.04	86.88	89.85	5	2
	BR6	92.99	86.83	89.81	0	0	92.76	86.52	89.53	0	0	92.70	85.31	88.86	2	0	92.79	87.17	89.89	6	3
	BR7	93.39	87.95	90.59	1	0	93.06	87.83	90.37	0	0	92.91	86.58	89.63	1	1	93.43	87.32	90.27	4	0
Strong heterogeneous	BR8	92.87	88.53	90.65	0	0	93.12	88.91	90.96	0	0	92.85	87.14	89.90	2	2	92.57	87.79	90.12	0	0
	BR9	91.93	89.39	90.64	0	0	92.38	89.87	91.11	0	0	92.31	88.62	90.43	0	0	92.83	88.41	90.57	3	2
	BR10	92.86	89.68	91.24	2	2	93.24	90.13	91.66	0	0	92.23	88.71	90.44	1	0	92.70	88.86	90.74	1	1
	BR11	91.61	88.94	90.25	1	0	92.29	89.62	90.93	0	0	91.77	88.91	90.32	0	0	92.65	89.26	90.92	2	1
	BR12	91.26	89.53	90.39	0	0	92.43	89.84	91.12	0	0	91.56	88.57	90.04	0	0	91.42	89.74	90.57	0	0
	BR13	91.47	89.12	90.28	1	0	92.34	89.66	90.98	0	0	91.11	88.23	89.65	0	0	92.17	89.85	91.00	1	0
	BR14	90.81	88.61	89.69	0	1	91.16	89.76	90.45	0	0	91.06	87.40	89.19	0	0	91.64	89.91	90.77	0	0
	BR15	90.47	89.27	89.87	2	2	90.73	89.58	90.15	0	0	90.46	87.95	89.19	0	0	91.23	89.47	90.34	0	0
Mean	AV.1–7	92.62	85.25	88.78	4	2	92.26	85.10	88.53	3	2	91.91	84.60	88.10	12	6	92.49	85.45	88.83	38	17
	AV.8–15	91.66	89.13	90.38	6	5	92.21	89.67	90.92	0	0	91.67	88.19	89.89	3	2	92.15	89.16	90.63	7	4
	AV.1–15	92.11	87.32	89.63	10	7	92.23	87.54	89.81	3	2	91.78	86.52	89.06	15	8	92.31	87.43	89.79	45	21

## Data Availability

The data used to support the findings of this study are available from the corresponding author upon request.
